# Identification of Bile Salt Hydrolase and Bile Salt Resistance in a Probiotic Bacterium *Lactobacillus gasseri* JCM1131^T^

**DOI:** 10.3390/microorganisms9051011

**Published:** 2021-05-08

**Authors:** Hiroyuki Kusada, Kana Morinaga, Hideyuki Tamaki

**Affiliations:** 1 Bioproduction Research Institute, National Institute of Advanced Industrial Science and Technology, Tsukuba, Ibaraki 305-8566, Japan; k.morinaga@aist.go.jp; 2 Faculty of Life and Environmental Sciences, University of Tsukuba, Tsukuba, Ibaraki 305-8572, Japan

**Keywords:** bile salt hydrolase, *Lactobacillus gasseri*, Ntn-hydrolase family protein, probiotics

## Abstract

*Lactobacillus gasseri* is one of the most likely probiotic candidates among many *Lactobacillus* species. Although bile salt resistance has been defined as an important criterion for selection of probiotic candidates since it allows probiotic bacteria to survive in the gut, both its capability and its related enzyme, bile salt hydrolase (BSH), in *L. gasseri* is still largely unknown. Here, we report that the well-known probiotic bacterium *L. gasseri* JCM1131^T^ possesses BSH activity and bile salt resistance capability. Indeed, this strain apparently showed BSH activity on the plate assay and highly tolerated the primary bile salts and even taurine-conjugated secondary bile salt. We further isolated a putative BSH enzyme (LagBSH) from strain JCM1131^T^ and characterized the enzymatic function. The purified LagBSH protein exhibited quite high deconjugation activity for taurocholic acid and taurochenodeoxycholic acid. The *lagBSH* gene was constitutively expressed in strain JCM1131^T^, suggesting that LagBSH likely contributes to bile salt resistance of the strain and may be associated with survival capability of strain JCM1131^T^ within the human intestine by bile detoxification. Thus, this study first demonstrated the bile salt resistance and its responsible enzyme (BSH) activity in strain JCM1131^T^, which further supports the importance of the typical lactic acid bacterium as probiotics.

## 1. Introduction

*Lactobacillus* species have been considered as one of the major targets of probiotic research. Several *Lactobacillus* species provide positive impacts on human health; symptomatic improvements by probiotics have been reported in cases of various hard-to-heal diseases, e.g., allergy [1], diarrhea [2], *Helicobacter pylori* infection [3], and irritable bowel syndrome [4]. These probiotic effects are generally strain-specific and differ depending on each strain even among *Lactobacillus* strains of same species [5,6,7,8,9]. Among the several criteria for selecting candidate probiotic strains of *Lactobacillus* spp., bile salt resistance is one of the most important selective criteria, since bile salts are well known as strong surfactants and bile exposure in gastrointestinal tract is intensely toxic for probiotic *Lactobacillus* species to survive and retain activity in human intestine [10,11].

Bile salt resistance is mainly provided by bile salt hydrolase (BSH, EC3.5.1.24), an enzyme that deconjugates glycine and/or taurine-conjugated bile salts [12], though other resistance mechanisms (i.e., efflux pumps, stress response proteins, and cell wall modifications) have been reported [13]. Genes encoding BSH have been found in various *Lactobacillus* species [14], and the number of *bsh* gene orthologs vary in accordance with species and strains [11,14,15,16,17]. BSH enzymes perform a crucial role in bile detoxification and thereby improve the colonization and survival of host probiotic bacteria in the human gastrointestinal tract [18]. In addition, BSH enzymes are known to involve in the reduction of blood cholesterol levels, the regulation of lipid absorption, glucose metabolism, and energy homeostasis in humans [19], which means that the bile salt deconjugation ability through the enzyme BSH has been widely recognized as a probiotic biomarker [20].

*Lactobacillus gasseri* type strain JCM1131^T^ is commensal, produces lactic acid, and is widely known as a typical probiotic bacterium. In fact, several probiotic properties of this strain have been reported [21,22,23]. However, it has remained largely unclear whether strain JCM1131^T^ has BSH activity and bile salt resistance capability. In the present study, we demonstrated the BSH activity and bile salt resistance ability in *L. gasseri* strain JCM1131^T^. We found the putative *bsh* gene in the genome and determined that its recombinant protein could functionally act as BSH mediating the bile salt resistance in the strain through molecular cloning, biochemical characterization, and transcriptional analyses.

## 2. Materials and Methods

### 2.1. Bacterial Strains Used in This Study

A probiotic lactic acid bacterium, *Lactobacillus gasseri* JCM1131^T^ (=DSM20243^T^=ATCC33323^T^), was obtained from the Japan Collection of Microorganisms (RIKEN BRC, Tsukuba, Japan). This strain was cultivated using Gifu anaerobic medium (GAM, Nissui Pharmaceutical Co., Ltd., Tokyo, Japan) and de Man–Rogosa–Sharpe medium (MRS, Difco Laboratories, Detroit, MI, USA) with headspace gas of N_2_/CO_2_ (80:20, *v*/*v*) at 37 °C under anaerobic conditions. *Escherichia coli* strain BL21 (DE3) Champion^TM^21 (SMOBIO Technologies, Hsinchu City, Taiwan) was used for heterologous expression experiments. *E. coli* was cultured in LB broth supplemented with 50 μg/mL kanamycin (FUJIFILM Wako Pure Chemical Corporation, Osaka, Japan) at 37 °C with shaking.

### 2.2. Cloning and Heterologous Expression of a Putative Bile Salt Hydrolase Gene

Based on the sequence analyses and homology searches using NCBI BLAST program (https://blast.ncbi.nlm.nih.gov/Blast.cgi) (accessed on 8 June 2020), UniProt BLAST tool (https://www.uniprot.org/blast/) (accessed on 8 June 2020), InterProScan (http://www.ebi.ac.uk/interpro/search/sequence-search) (accessed on 8 June 2020), and Pfam (http://pfam.xfam.org/) (accessed on 8 June 2020), we screened a gene encoding putative BSH from the complete genome sequence of strain JCM1131^T^ (accession number CP000413). A putative *bsh* gene (named as *lagBSH*) was commercially synthesized with codon optimization for heterologous expression in *E. coli* (GenScript, Piscataway, NJ, USA). The *lagBSH* gene was subcloned into the NdeI and EcoRI sites of pET28-b (Novagen, Madison, WI, USA) expression vector.

The heterologous gene expression and protein purification experiments were performed according to our previous study with slight modifications [24]. In brief, the constructed plasmid was transformed into *E. coli* BL21 (DE3) Champion^TM^21 competent cells and *E. coli* strain was cultured on LB broth at 37 °C until OD_600_ reached 0.4–0.6. Isopropyl-β-D-thiogalactopyranoside (IPTG, Nacalai Tesque, Kyoto, Japan) was added at the final concentration of 100 μM to the culture medium. After adding of IPTG, the *E. coli* cells were incubated at 20 °C for overnight with shaking. The cells were harvested by centrifugation at 5800× *g* for 10 min, suspended in buffer (20 mM Tris, 150 mM NaCl, 5% glycerol, 5 mM imidazole, pH 7.5), and disrupted for 5 min by sonication using an ultrasonic disintegrator (Sonicator Branson Sonifier 250 (Branson, Danbury, CT, USA); output control: 5, duty cycle: 50) in an ice-water bath. The cell debris were removed by centrifugation and the resulting supernatant was mixed with Ni-NTA Agarose HP (FUJIFILM Wako Pure Chemical Corporation). The His_6_-tagged recombinant protein was washed and eluted according to the previous study [24]. The eluted fraction was further dialyzed with buffer (20 mM Tris, 150 mM NaCl, 5% glycerol) using semipermeable membrane (Spectra/Por 3 membrane MWCO: 3500, Repligen, Waltham, MA, USA) and concentrated using Amicon Ultra centrifugal filter devices (30,000 MWCO, Millipore, Billerica, MA, USA). The purified protein was treated with sample buffer (Bio-Rad, Hercules, CA, USA), heat-denatured at 95 °C for 5 min, and analyzed on sodium dodecyl sulfate polyacrylamide gel electrophoresis (SDS-PAGE) using 12% Mini-PROTEAN TGX precast polyacrylamide gel (Bio-Rad) [24]. The gel was stained with QC Colloidal Coomassie Stain (Bio-Rad) through gentle agitation.

### 2.3. Bile Salt Hydrolase Activity

The bile salt hydrolyzing activity of purified LagBSH was determined as described previously [25]. In brief, purified protein was mixed with 0.24 mg/100 μL of conjugated bile salts (glycocholic acid (GCA, Sigma-Aldrich, St. Louis, MO, USA), glycodeoxycholic acid (GDCA, Sigma-Aldrich), taurocholic acid (TCA, Nacalai Tesque), taurochenodeoxycholic acid (TCDCA, Sigma-Aldrich), and taurodeoxycholic acid (TDCA, Nacalai Tesque)) and incubated at 37 °C. The reaction was stopped by adding 15% trichloroacetic acid (FUJIFILM Wako Pure Chemical Corporation) and the resulting solution was centrifuged at 10,000× *g* for 15 min at 20 °C. The supernatant was then mixed with 0.3 M borate buffer with 1% SDS (pH 9.5), and 0.3% 2,4,6-trinitrobenzenesulfonic acid solution (Tokyo Kasei Kogyo Co., Ltd., Tokyo, Japan). The resulting mixture was incubated for 30 min at room temperature under dark condition, and then 0.6 mM HCl was added to stop the reaction. The absorbance at 416 nm was measured using a SPARK 10M multimode microplate reader (TECAN, Männedorf, Switzerland). The assays were performed in eight replicates. Student’s *t*-test was used to assess the presence of statistically significant differences (α = 0.05) using GraphPad Prism version 8.0 software program (GraphPad Software, San Diego, CA, USA). As a negative control, a bile salt solution was reacted with buffer instead of purified protein.

### 2.4. Biochemical Characterization

The optimum pH and temperatures of LagBSH were determined according to the methods described [20] with slight modifications as follows. The purified LagBSH protein was mixed with taurocholic acid (TCA) at selected temperatures (10–90 °C, in intervals of 10 °C) and pH (pH 3.0–pH 10.0, in intervals of pH 1.0) ranges. To determine the effects of pH on enzyme activity of LagBSH, we used various Good’s buffer solution based on the pH range (acetate buffer [CH_3_COONa·3H_2_O] pH 3.0–4.0; MES buffer [C_6_H_13_NO_4_S·H_2_O] pH 5.0–6.0; HEPES buffer [C_8_H_18_N_2_O_4_S] pH 7.0–8.0; CAPS [C_9_H_19_NO_3_S] pH9.0–10.0). After incubation for 6 h, the released taurine was detected as described above. All experiments were performed in eight replicates.

### 2.5. Bile Salt Tolerance Test

The bile salt tolerance ability of *Lactobacillus gasseri* JCM1131^T^ was estimated and calculated from its survival rates according to the previous study [26]. In brief, a full-grown culture of strain JCM1131^T^ was mixed with GCA, GDCA, TCA, and TDCA at final concentrations of 0.05%. Cells were anaerobically incubated at 37 °C for 6 h, and the optimal densities (OD_600_) were measured every hour using an Ultrospec 500 Pro visible spectrophotometer (GE Healthcare Life Sciences, Buckinghamshire, UK). As a negative control, strain JCM1131^T^ was incubated in GAM medium without bile salt. The assays were performed in triplicates.

Minimum inhibitory concentrations (MICs) were determined as the lowest concentration of bile salts preventing visible growth of *L. gasseri* JCM1131^T^ on MRS agar. Strain JCM1131^T^ was cultivated in MRS broth and inoculated on MRS agar plate with a selected bile salt. Plates were anaerobically incubated at 37 °C for 5 days. The tested bile salts were GCA, GDCA, TCA, and TDCA at final concentrations of 0.01%, 0.05%, 0.1%, 0.25%, and 0.5%. All experiments were carried out in triplicates.

### 2.6. Structural Modeling

Three-dimensional conformations of LagBSH were predicted using Swiss-Model workspace (https://swissmodel.expasy.org/) (accessed on 8 June 2020). [27]. The superposition analyses were performed and visualized using UCSF Chimera software [28]. Crystal structure of a known BSH enzyme, CpBSH from *Clostridium perfringens* 13 [29], was provided by Protein Data Bank (http://www.rcsb.org/pdb/home/home.do) (accessed on 8 June 2020).

### 2.7. Transcriptional Analysis

Reverse transcription polymerase chain reaction (RT-PCR) analyses of the *lagBSH* gene were performed as follows. Strain JCM1131^T^ was cultured on MRS broth with or without TCA and TDCA at final concentration of 0.05%. The total RNA samples were isolated using the RNeasy Mini Kit (Qiagen, Germantown, MD, USA), and the resulting RNA samples were treated with TURBO™ DNase (Thermo Fisher Scientific, Waltham, MA, USA) to remove contaminated genomic DNA. The presence of chromosomal genomic DNA was confirmed by PCR analysis with the 16S rRNA gene universal PCR primers 530F and 907R using each RNA samples as template. Reverse transcription reactions were performed using SuperScript IV Reverse Transcriptase (Thermo Fisher Scientific) in a 25 μL reaction volume according to the manufacturer’s instruction. The synthesized cDNA samples were used as the PCR template with the following two PCR primer sets: *lagBSH*-Aset (5’-TCACACCACGCAACTATCCTC-3’ and 5’-GTTGCCAAGGTTAGTAAGATGCC-3’, amplicon size: 467 bp) and *lagBSH*-Bset (5’-TTAGCTTCTTACGAAATTATGC-3’ and 5’-GAATGCTATCACCTGGTAAAC-3’, amplicon size: 376 bp). The PCR products were analyzed using agarose gel electrophoresis in 2.0% agarose and were stained with Gelred (Fujifilm Wako Pure Chemical Corporation).

## 3. Results and Discussion

### 3.1. Identification of BSH Activity and Bile Salt Resistance of Lactobacillus gasseri JCM1131^T^

**Figure 1:**
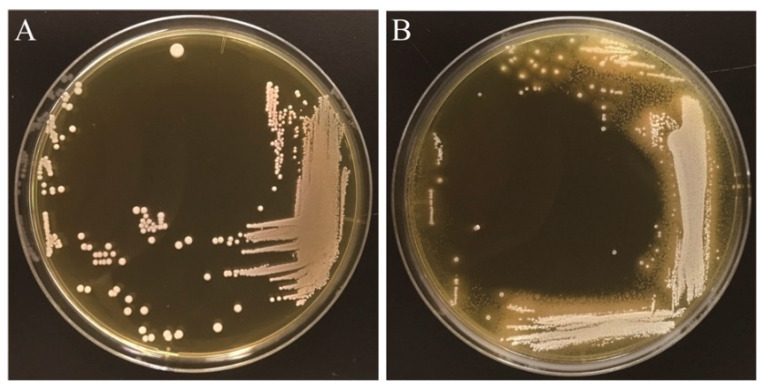
Bile salt hydrolase activity in *Lactobacillus gasseri* JCM1131^T^. Full-grown culture of *L. gasseri* JCM1131^T^ was streaked on an MRS agar plate (**A**) or an MRS agar plate supplemented with 0.25% taurodeoxycholic acid (**B**). The plates were anaerobically incubated at 37 °C for 5 days. The visible halo surrounding colonies and the white precipitates with colonies are the well-known indicator of bacterial BSH activity [19,30].

In the present study, we first investigated whether *L. gasseri* JCM1131^T^ shows BSH activity using the standard plate assay method. We observed that the visible halo surrounding colonies and the white precipitates with colonies when strain JCM1131^T^ was cultured on an MRS agar plate supplemented with taurodeoxycholic acid (TDCA), one of the major conjugated bile salts in human gastrointestinal tract (Figure 1). These characteristics (i.e., halo and white precipitates) are the well-known indicators of BSH activity [19,30], clearly suggesting that strain JCM1131^T^ represents BSH activity, though the previous study reported that *L. gasseri* ATCC33323^T^ (=JCM1131^T^) showed no significant BSH activity [31]. Importantly, Allain et al. demonstrated that *L. gasseri* strain CNCM I-4884 with strong BSH activity exhibited significant antiparasitic ability that antagonizes growth of the most common waterborne parasite (*Giardia*) [32] and further revealed that the antiparasitic effects of *Lactobacillus* spp. were well correlated with the expression of BSH activities [32]. Based on this fact, we expect that *L. gasseri* JCM1131^T^ with BSH activity may also exhibit antiparasitic activity as well as strain CNCM I-4884, though future study needs to clarify this point.

**Figure 2:**
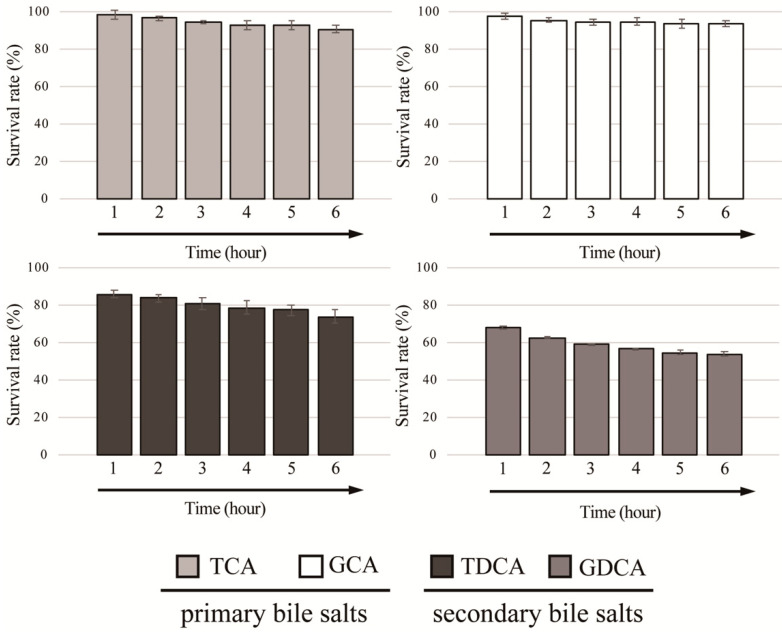
Bile salt tolerance activity in *Lactobacillus gasseri* JCM1131^T^. Full-grown culture of strain JCM1131^T^ was mixed with GCA, GDCA, TCA, and TDCA at final concentrations of 0.05% and incubated anaerobically at 37 °C. The optimal density (OD_600_) was measured every hour and survival rates were calculated as described previously [26]. The survival rate of control (without bile salt) was defined as 100%. Results indicated mean ± SD obtained in triplicate experiments.

We further determined the survivability of *L. gasseri* JCM1131^T^ against four different conjugated bile salts (GCA, GDCA, TCA, and TDCA) at final concentration of 0.05%. As shown in Figure 2, strain JCM1131^T^ showed high survivability toward primary bile salts (TCA and GCA) and the survival rates of strain JCM1131^T^ against TCA and GCA reached above 90% after exposed to the bile salts for 6 h. Additionally, this strain also exhibited moderate and low survivability toward TDCA and GDCA (secondary bile salts), and the survival rates against TDCA and GDCA were above 70% and below 60%, respectively (Figure 2).

We further investigated the bile salt tolerance capacity of *L. gasseri* JCM1131^T^ by determining the minimum inhibitory concentrations (MICs). As shown in Table 1, this strain showed low MIC value (0.05%) to GDCA, indicating that GDCA is toxic to strain JCM1131^T^. De Smet et al. suggested that the high toxicity of GDCA would be caused by its weak acid property (TDCA is strong acid property) [33]. They further hypothesized that the protonated form of bile salts exhibited toxicity as it imported protons in the cell [33]. This hypothesis seems to be reasonable since weak acids are pretty much easier to protonate than strong acids. However, strain JCM1131^T^ displayed a higher resistance ability (MICs were >0.5%) to a secondary bile salt (TDCA) as well as the primary bile salts (TCA and GCA) (Table 1), despite the fact that secondary bile salts have been known to be more toxic than primary bile salts [34]. Since the average bile concentration in human intestine has been estimated to be 0.3% w/v [35], our findings suggest that strain JCM1131^T^ could have bile salt tolerance ability toward TCA, GCA, and TDCA. Previous studies reported that other strains of *L. gasseri* (BGHO89, 4M13, and FR4) showed high bile salt resistance ability (toward more than 0.3% bile salts) [36,37,38], suggesting that gut-derived *L. gasseri* strains would generally have bile salt resistance ability to survive and colonize the mammalian digestive tracts. Interestingly, these *L. gasseri* strains (BGHO89, 4M13, and FR4) with high bile salt resistance capacity further exhibited some probiotic functions including acid tolerance, bacteriocin production, antioxidation, and cholesterol-lowering activity [36,37,38]. Thus, although it has been reported that some other strains of *L. gasseri* show bile salt tolerance so far [21,36,37], the correlations between their bile salt tolerance ability and BSH activity have not been well demonstrated. In the present study, we first revealed both bile salt tolerance capability and its related key enzymatic function (BSH activity) in *L. gasseri* JCM1131^T^, and these findings provide additional insights into the probiotic function in a well-known representative of the probiotic lactic acid bacterium.

### 3.2. Sequence and Phylogenetic Analyses of a Putative BSH Gene

**Figure 3:**
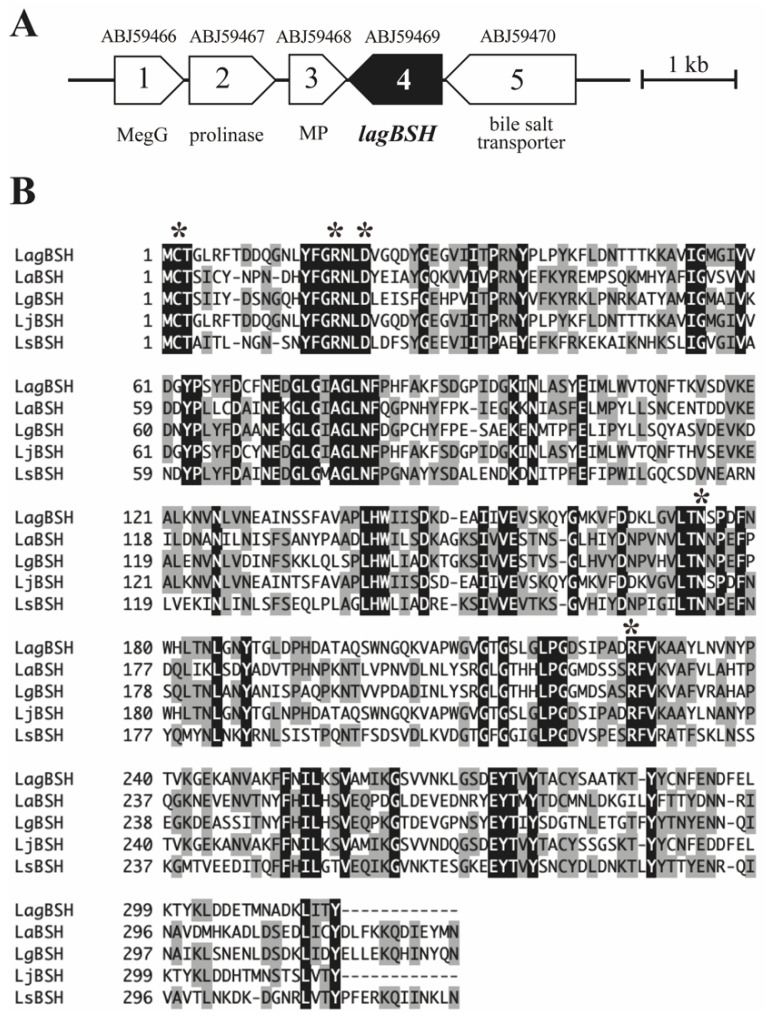
(**A**) Physical map of the predicted *bsh* gene on the genome sequence of *Lactobacillus gasseri* JCM1131^T^ (accession number CP000413). The scale bar indicates a 1 kb length of nucleotide. A putative *bsh* gene (*lagBSH*) and its surrounding ORFs are represented by filled and open symbols, respectively. Brief annotation and protein ID were provided. MegG, demethylmenaquinone methyltransferase; MP, membrane protein. (**B**) Multiple alignment of amino acid sequences of BSHs. Amino acid sequence of LagBSH was aligned and compared with known BSHs from *Lactobacillus* species. The black and gray shading indicates identical and similar amino acid residues, respectively. The conserved residues (Cys, Arg, Asp, Asn, and Arg) relevant to the predicted active site are indicated by black asterisks. Abbreviations: LaBSH (AAV42923) from *Lactobacillus acidophilus* NCFM; LgBSH (WP_020806888) from *Lactobacillus gasseri* FR4; LjBSH (AAC34381) from *Lactobacillus johnsonii* 100-100; LsBSH (JX120368) from *Lactobacillus salivarius* B-30514.

Since both BSH activity and bile salt resistance capability of *L. gasseri* JCM1131^T^ were revealed, we then performed cloning and heterologous expression of the gene candidates associated with BSH activity. We herein found a putative bile salt hydrolase gene (designated as *lagBSH*) in *L. gasseri* JCM1131^T^ genome (CP000413) based on the sequence analyses and homology searches (Figure 3A). The putative *lagBSH* gene comprises 951 bp. The deduced amino acid sequence of LagBSH (316 amino acids) was related to the cholylglycine hydrolase family of the Ntn-hydrolase superfamily proteins based on the domain and sequence comparison. The multiple amino acid sequence alignments revealed that LagBSH protein shared five residues (Cys, Arg, Asp, Asn, and Arg) associated with active site with previously identified BSHs from *Lactobacillus* species (Figure 3B). Three-dimensional superposition analyses further revealed that the overall structure of LagBSH is composed of well-known αββα-sandwich folds of cholylglycine hydrolase proteins (Supplementary Figure S1A), which are similar to the structure of CpBSH, BSH from *Clostridium perfringens* 13 [29]. The putative LagBSH further conserved the catalytic active site structure identified with CpBSH (Supplementary Figure S1B). It has been reported in previous studies that N-terminal cysteine residue (Cys-2) plays a critical role in the BSH activity as catalytic nucleophile [29], and thus the putative protein would function as the BSH enzyme.

**Figure 4:**
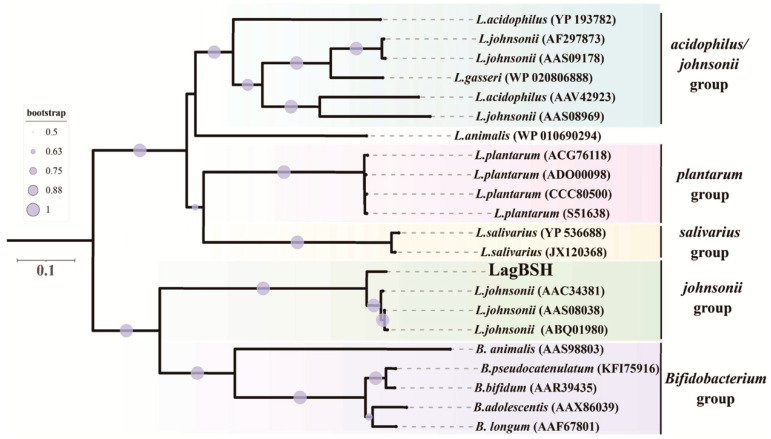
Phylogenetic analysis of LagBSH with cholylglycine hydrolase family proteins. The phylogenetic tree was constructed with MEGA X software using the neighbor-joining method (1000 bootstrap replications) [39]. Bootstrap values greater than 50% are shown by circle symbols whose size correlates with the bootstrap values. CpBSH, BSH from *Clostridium perfringens* 13, was used as an outgroup.

Amino acid sequence comparison analyses using the standard BLASTP protein–protein BLAST search revealed that LagBSH exhibited high similarity (~93.99% amino acid sequence homology) to known BSHs, especially BSHs from *L. johnsonii* strain 100-100 (93.99%), strain NCC533 (93.67%), and strain PF01 (93.35%) (Supplementary Table S1). LagBSH exhibited significantly lower similarity to LgBSH form *L. gasseri* FR4 (39.94%) [20], despite the fact that both BSH enzymes are commonly derived from *L. gasseri*. The phylogenetic analysis demonstrated that the cholylglycine hydrolase family proteins were subdivided into several groups (Figure 4), and we found that LagBSH was classified into the *L.* *johnsonii* BSH subgroup (Figure 4). LgBSH was categorized into the *L. acidophilus*/*johnsonii* BSH subgroup, indicating that LagBSH are phylogenetically distinct from LgBSH. These sequence, structural, and phylogenetic analyses further suggested that LagBSH would have BSH activity as well as known BSHs from other *Lactobacillus* species.

### 3.3. Heterologous Expression of the Putative BSH Gene in E. coli

To obtain the recombinant protein, *lagBSH* gene was commercially synthesized with codon optimization for heterologous expression in *Escherichia coli* and subcloned into the NdeI and EcoRI sites of pET28-b expression vector. The gene was overexpressed in *E. coli* BL21 (DE3) Champion^TM^21 and recombinant protein was purified by Ni-affinity chromatography. Based on sodium dodecyl sulfate polyacrylamide gel electrophoresis (SDS-PAGE) analysis, the molecular weight of purified His_6_-LagBSH protein was approximately 35.0 kDa in size (Supplementary Figure S2), which is nearly identical with the theoretical molecular weight based on its amino acid sequence.

### 3.4. Bile Salt Hydrolase Assay

**Figure 5:**
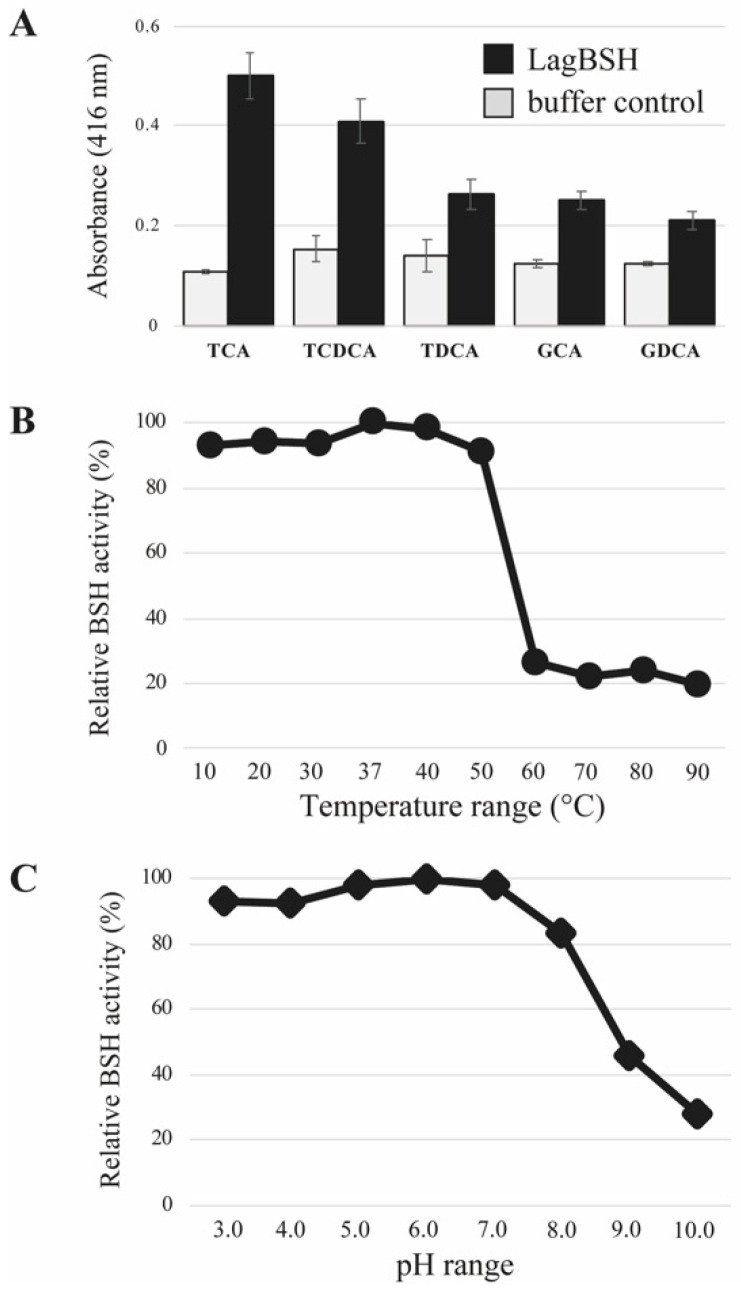
Bile salt hydrolase activity and biochemical characterization of LagBSH. (**A**) BSH activities were measured toward five human bile salts: glycocholicacid (GCA), glycodeoxycholic acid (GDCA), taurocholic acid (TCA), taurodeoxycholic acid (TDCA), and taurochenodeoxycholic acid (TCDCA). Values are indicated as means for eight technical experiments (*n* = 8). Error bars represent standard deviation (SD). (**B**) Effect of temperature (10–90 °C) and (**C**) pH (pH 3.0–pH 10.0) on BSH activity toward TCA of LagBSH. Each value is expressed as means for eight technical replicates (*n* = 8). Maximum activity was taken as 100%.

The bile salt hydrolyzing activity of the purified LagBSH was determined by detecting the released glycine or taurine from conjugated bile salts as described previously [25]. We selected five major mammalian conjugated bile salts (TCA, TDCA, TCDCA, GCA, and GDCA) as substrates. The recombinant LagBSH clearly exhibited significant BSH activity toward all substrates tested. In particular, as shown in Figure 5A, LagBSH showed its high activity toward TCA and TCDCA. The BSH activities toward the other three substrates (TDCA, GCA, and GDCA) are relatively low, suggesting that LagBSH is a functional BSH enzyme, particularly showing high specificity for taurine-conjugated bile salts (TCA and TCDCA). Such substrate specificity of LagBSH was consistent with the previous studies. In fact, the previously identified BSH from *L. johnsonii* PF01 sharing high homology with LagBSH (93.35% homology) exhibited deconjugation activity against taurine-conjugated bile salts, but not glycine-conjugated bile salts [40], though most BSHs from lactic acid bacteria are more likely to deconjugate glycine-conjugated bile salts rather than taurine-conjugated bile salts [40]. LgBSH from *L. gasseri* FR4 showed higher BSH activity toward glycine-conjugated bile salts than taurine-conjugated ones [20], indicating that the substrate specificity were also quite different between LagBSH and LgBSH, even though both enzymes are derived from the same species, *L. gasseri*. These enzymatic characteristics agree well with the previous report that the substrate specificity of BSH enzymes may be strain-specific [41]. Further structural and site-directed mutagenesis analyses of LagBSH would perhaps lead to a better understanding of its substrate preference. In total, LagBSH has apparent BSH activity, and this functional enzyme would confer bile detoxification on the host microorganism *L. gasseri* JCM1131^T^.

### 3.5. Biochemical Characterization of LagBSH

The optimum temperature and pH of LagBSH were determined. The purified LagBSH protein was mixed with taurocholic acid (TCA) at selected temperature (10–90 °C, in intervals of 10 °C) and pH (pH 3.0–pH 10.0, in intervals of pH 1.0) ranges. The maximum BSH activity was observed at 37 °C (Figure 5B). We confirmed that LagBSH exhibited high BSH activity in wide temperature range (at 10–50 °C) and it retained above 80% of its original activity, whereas the enzyme activity significantly declined with higher temperature (>60 °C) (Figure 5B). In addition, the maximum BSH activity of LagBSH was observed at pH 6.0 (Figure 5C). LagBSH exhibited stable activity and retained approximately above 80% of their original activity at broad pH range (pH 3.0–8.0), whereas significant decreases in enzyme activities were observed at more than or equal to pH 9.0 (Figure 5C). The optimum temperature and pH of LagBSH (37 °C and pH 6.0) are highly consistent with conditions of the human small intestine (around 37 °C and pH 5.0–8.0) and the growth condition of strain JCM1131^T^ in MRS broth (pH 6.0–6.5, 37 °C) according to the website of the Japan Collection of Microorganisms (https://jcm.brc.riken.jp/en/) (accessed on 8 June 2020). These biochemical features of LagBSH further support our hypothesis that this enzyme may contribute to bile detoxification of *L. gasseri* JCM1131^T^.

### 3.6. Transcriptional Analysis of lagBSH Gene

**Figure 6:**
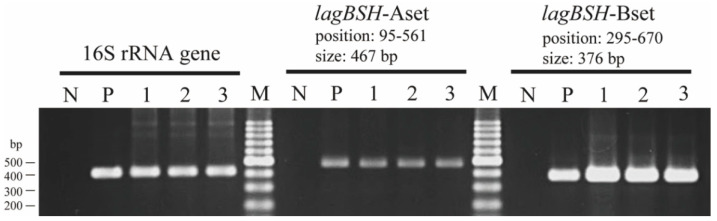
RT-PCR analyses of *lagBSH* genes in *L. gasseri* JCM1131^T^. Sterilized water (lane N) and genomic DNA of strain JCM1131^T^ (lane P) were used as negative and positive control, respectively. The products of reverse transcription from total RNA of nonsupplemented (lane 1), TCA-supplemented (lane 2), and TDCA-supplemented (lane 3) strain JCM1131^T^ cells were used as the template for PCR, respectively. The 16S rRNA gene (378 bp) was used as internal standard control. Lane M, molecular size markers (100 bp DNA ladder, Promega, Madison, WI, USA).

To determine the regulation of gene transcription of the *lagBSH* gene, reverse transcription polymerase chain reaction (RT-PCR) analyses were conducted. We found that the *lagBSH* gene was constituently expressed in *L. gasseri* JCM1131^T^ (Figure 6, lane 1). In addition, the *lagBSH* gene transcription was also observed in this strain exposed to TCA (Figure 6, lane 2) and TDCA (Figure 6, lane 3), suggesting that the exposure to TCA and TDCA may have little effect on the *lagBSH* gene transcription in strain JCM1131^T^. Since bile salt concentrations reach the millimolar level in the human small intestine and it should be toxic to the intestinal bacteria [12], strain JCM1131^T^ seems to constantly produce LagBSH enzyme to tolerate high concentration of bile salts and survive in the gut.

## 4. Conclusions

In this study, we identified that *Lactobacillus gasseri* JCM1131^T^ displayed bile salt resistance capacity toward primary bile salts and taurine-conjugated secondary bile salt. The present study further demonstrated that strain JCM1131^T^ exhibited apparent BSH activity, although this strain has been considered to be a non-BSH-producer so far. Moreover, we clarified the correlations between bile salt resistance and BSH activity in *L. gasseri*, which has been rarely investigated and poorly understood; indeed, only two strains (*L. gasseri* FR4 and *L. gasseri* CNCM I-4884 isolated from chicken and carious tooth, respectively [20,32]) have been reported to show both bile salt resistance ability and BSH activity by producing their BSH enzymes (LgBSH isolated from strain FR4 [20]) among *L. gasseri* isolates. In the present study, we also found that BSH enzyme from *L. gasseri* JCM1131^T^ (LagBSH) was significantly different from LgBSH in terms of their amino acid sequence homology, substrate specificity, and phylogenetic position. Since strain JCM1131^T^ is a human-derived lactic acid bacterium that exhibits oxalate-degradation activity [23] and increases in interleukin-10 production [22], this study could further expand and deepen the understanding of this beneficial probiotic bacterium.

In addition, we performed the enzymatic, transcriptional, and phylogenetic characterization of LagBSH isolated from strain JCM1131^T^. LagBSH could function as BSH enzyme able to hydrolyze conjugated bile salts especially against taurocholic acid and taurochenodeoxycholic acid. We further demonstrated that the *lagBSH* gene was constantly transcribed in *L. gasseri* JCM1131^T^. Therefore, this functional enzyme would confer a survival advantage on strain JCM1131^T^ within the human intestine by bile detoxification. Because BSH activity exert further positive effects on human health such as weight loss and cholesterol lowering [19], future studies need to examine the probiotic effects related to the BSH activity of *L. gasseri* JCM1131^T^ by in vivo animal model study. Altogether, our findings provide additional insights into the probiotic function in a well-known representative of probiotic lactic acid bacterium *L. gasseri* JCM1131^T^.

## Figures and Tables

**Table 1:** Minimum inhibitory concentrations of bile salts against strain JCM1131^T a^.

Strain	Minimum Inhibitory Concentrations (%)			
	Substrates			
	TCA	TDCA	GCA	GDCA
JCM1131^T^	>0.5	>0.5	>0.5	0.05

^a^ TCA, taurocholic acid; TDCA, taurodeoxycholic acid; GCA, glycocholic acid; GDCA, glycodeoxycholic acid. The tested concentrations of bile salts were 0.01, 0.05, 0.1, 0.25, and 0.5%.

## Data Availability

Not applicable.

## Supplementary Materials

The following are available online at https://www.mdpi.com/article/10.3390/microorganisms9051011/s1, Figure S1: Structural analyses of LagBSH, Figure S2: SDS-PAGE analysis of purified LagBSH, Table S1, The BSH sequence homology among *Lactobacillus* species.
