# Supplementary material for: Identification of Bile Salt Hydrolase and Bile Salt Resistance in a Probiotic Bacterium Lactobacillus gasseri JCM1131T
Source: Microorganisms. 2021 May 8;9(5):1011. doi: 10.3390/microorganisms9051011 (PMC8151060; doi:10.3390/microorganisms9051011)
Supplement: Supplementary file 1 [file microorganisms-09-01011-s001.zip › microorganisms-1201198-supplementary.pdf]

Supplementary Materials

Identification of Bile Salt Hydrolase and Bile Salt Resistance in a Probiotic Bacterium *Lactobacillus gasseri* JCM1131^T

Hiroyuki Kusada [1,*], Kana Morinaga [1] and Hideyuki Tamaki [1,2,*]

[1] Bioproduction Research Institute, National Institute of Advanced Industrial Science and Technology, Tsukuba, Ibaraki, 305-8566, Japan; k.morinaga@aist.go.jp
[2] Faculty of Life and Environmental Sciences, University of Tsukuba, Tsukuba, Ibaraki, 305-8572, Japan
* Correspondence: kusada-hiroyuki@aist.go.jp (H.K.); tamaki-hideyuki@aist.go.jp (H.T.); Tel.: +81-29-861-6591 (H.K.); +81-29-861-6592 (H.T.); Fax: +81-29-861-6587 (H.K. & H.T.)

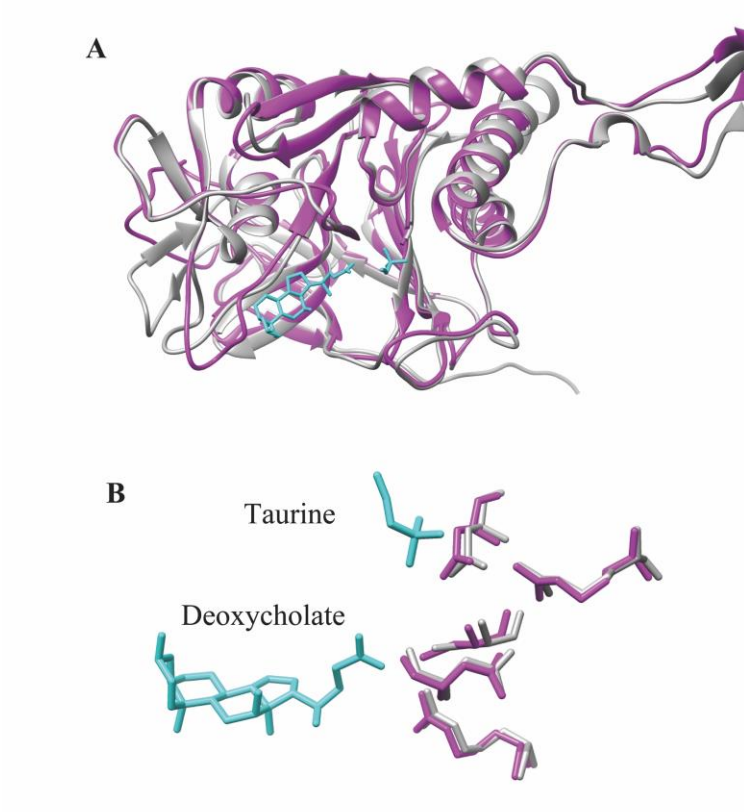

Figure S1. Structural analyses of LagBSH. Overall (A) and active site (B) structural superposition of LagBSH (magenta) with CpBSH from *Clostridium perfringens* 13 (light gray; PDB entry 2BJF). Taurine and deoxycholate molecules were shown in cyan stick.

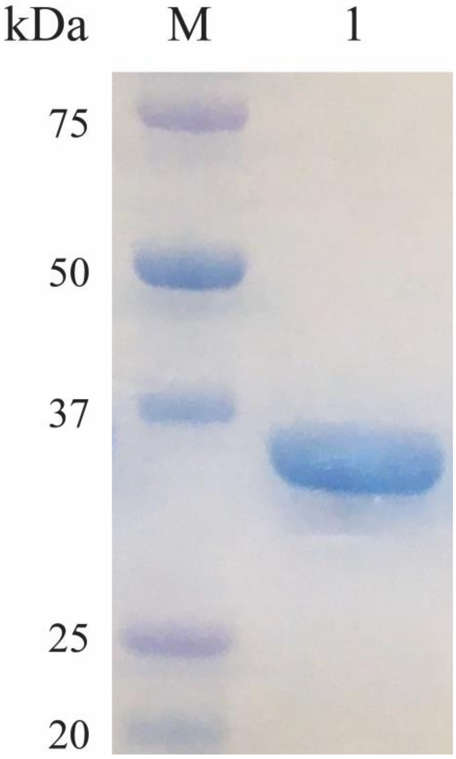

Figure S2. SDS-PAGE analysis of purified LagBSH. After nickel affinity chromatography, purified protein was loaded on 12% SDS-PAGE gel (Bio-Rad). Lane M, molecular size-marker (Precision Plus Protein™ Dual Color Standards, 10-250 kDa); lane 1, LagBSH.

Table S1. The BSH sequence homology among *Lactobacillus* species.

Species	Strain	Accession No	Amino acid sequence homology (%)
			LagBSH
L. acidophilus	**NCFM**	YP_193782	33.33
L. acidophilus	NCFM	AAV42923	35.00
L. animalis	unknown	WP_010690294	37.30
L. buchneri	NRRL B-30929	AEB72500	29.69
L. fermentum	NCDO 394	AEZ06356	29.06
L.gasseri	FR4	WP_020806888	39.94
L. johnsonii	PF01	ABQ01980	93.35
L. johnsonii	100-100	AF297873	39.20
L. johnsonii	100-100	AAC34381	93.99
L. johnsonii	NCC533	AAS09178	38.58
L. johnsonii	NCC533	AAS08969	32.91
L. johnsonii	NCC533	AAS08038	93.67
L. plantarum	MBUL69	ACG76118	38.05
L. plantarum	WCFS1	CCC80500	37.74
L. plantarum	LP80	S51638	37.74
L. plantarum	ST-III	ADO00098	37.74
L. plantarum	ST-III	ADN97280	30.32
L. plantarum	ST-III	ADN99975	30.67
L. plantarum	ST-III	ADN99333	27.84
L. reuteri	100-23	EDX41535	36.62
L. salivarius	UCC118	YP_536688	37.07
L. salivarius	NRRL B-30514	JX120368	37.38
L. salivarius	JCM1046	ACL98204	37.03
